# Evaluation and Enhancement of Triaging Services Through Quality Improvement Tools at Primary Health Care Level: A Clinical Audit Study

**DOI:** 10.1177/21501319231202204

**Published:** 2023-10-14

**Authors:** Ansif Pallath Majeed, Noora Alkubaisi, Kiran Harikumar, Hanan Al Mujalli, Abdul Ali Shah, Sharifullah Khan, Soraimah Sarip Socor

**Affiliations:** 1 Primary Health Care Corporation, Doha, Qatar

**Keywords:** primary care, triage, emergency visits, process map, quality improvement

## Abstract

**Background:**

The effective and efficient operation of emergency services at healthcare depends on triage decisions. Successfully implementing a triage system improves patient care, communication, and self-assurance.

**Methods:**

A baseline audit was conducted by reviewing a sample of 554 triage health records in September 2021. Many gaps were identified in the practice, and action plans were developed for improving it. Following the implementation of the action plan, a re-audit was conducted in September 2022 with a sample of 470 medical records.

**Results:**

Evidence suggested that nurses had made progress in correctly allocating the medical emergency triage category from 63% at baseline to 90% at the reaudit. The over-triage decreased in accordance with this adjustment, from 37% to 10%. Compliance with the suggested time target of 5 minutes for physicians to attend medical emergencies has shown a small improvement from 48% at baseline to 55% in the re-audit. Similar improvements were demonstrated in the other triage categories.

**Conclusion:**

A problem may have several causes, and since it is impossible to address every one of them, prioritizing the causes is usually the best course of action. Inadequate triage classification by nurses was one of the key reasons for the delay in physician appointment times in triage clinics. Triage nurses’ abilities should be enhanced to make this triage judgment. The audit team suggested that nurses should be given problem-based training, which will enhance the entire triage procedure.

## Introduction

In Qatar, primary healthcare facilities get most patient visits, which can range in age from children to the elderly. Triage has been served as a process of categorizing these patients according to their medical needs, regardless of order of arrival or factors such as gender, age, socioeconomic status, insurance coverage, immigration status, nationality, race, ethnicity, or religion. Triage is the first opportunity to immediately identify high-risk patients.^1^ Triage involves prioritization assessment of patients requiring urgent care and those with non-urgent conditions requiring more appropriate medical care, according to clinical severity and time urgency. The waiting time for consultation is one of the key metrics tracked at emergency department as a surrogate marker of timeliness and care quality.^2^ Treatment and resources must be prioritized for those with the greatest and most pressing medical needs. This allows medical staff to provide medical assistance to everyone in the facility most efficiently and productively. Preventive services including routine screenings for adults, children, and women can be given more time. Additionally, it aids in extending visitations for patients who require urgent care.^3^

The triage decision-making process is influenced by 3 interrelated factors: patient characteristics, the triage decision-maker, and the healthcare environment. Clinical judgment is frequently used during the triage execution process, which leads to high inter-rater variability and subpar predictive power.^1^ Triage is important in redistributing and shortening waiting times, increasing efficiency and effectiveness, increasing patient and family satisfaction, increasing the quality of health care, organizing financial matters, and evaluating the effectiveness of activities. Skillfully applied triage techniques can make the difference between quick, convenient, and cost-effective treatment.^4^

In primary health care, health centers offer patients a walk-in clinic where the first contact is with a nurse, thus it provides space for community nurse to use her expertise and skills in assessment and provision of lifestyle and self-care advise to the patients, after triage, she/he sends the patient to the correct treatment area. Research shows that nurse-led triage reduces patient visits to doctors without increasing mortality.^3^ Overcrowded clinics prevent patients from accessing doctors, putting patient safety at risk. Nurse-led triage in primary care can lead to safer and more efficient health care when patients whose health care needs are not assessed do not have to wait long to see a doctor. Triage leads to more patients being directed directly to appropriate care areas.^5^

Primary Health Care Corporation (PHCC) is the largest primary care organization in Qatar, having 31 established health centers across Qatar. The PHCC health centers offer 2 different types of appointments: walk-in clinics, which are regarded as the major entry point into the healthcare system, and appointments that must be scheduled in advance. Regular and urgent cases both use walk-in clinic facilities. Because not all patients can receive immediate or simultaneous care and only a minority of patients have life-threatening or urgent medical issues, it is difficult to predict the volume of attendance to a walk-in clinic. Therefore, individuals with injuries or illnesses that endanger their lives must be carefully detected as soon as they arrive. Thus, it is crucial to prioritize these patients since there may be instances where the volume of patients surpasses the capacity of the system, leading to chaos and congestion that may put patients’ safety at risk. The current PHCC policy that governs the Walk-In triages embodies 3 triage categories: medical emergency, medical priority, and medical routine. It suggests that patients assigned to the medical routine category should be treated by a doctor during the same shift on the same day, patients assigned to the medical priority category should be seen by a doctor within 90 min, and patients assigned to the medical emergency category should be examined by a doctor within 5 min. Despite concentrating on primary care, PHCC also manages emergency cases. Primary healthcare facilities in Qatar are conveniently located as patients with any clinical condition can avail the services of the Primary Healthcare Corporation. In other cases, these medical facilities must act as emergency clinics where a designated Code Blue team is available and ready to receive, respond, and manage emergency circumstances, with further stabilization the patient is being directed to secondary care. Therefore, it is essential in such situation to identify emergency cases by suitable categorization. In triaging, the first encounter of the patient is with the triage nurse, where categorization is based on assessment of clinical presentation. A proper categorization will likely improve the quality of patient services and could reduce mortality. Under-triaging and Over-triaging are 2 major errors that occur in the triaging process. Over-triaging is a measure that overestimates patient priority and assigns a higher category than required, while Under-triaging is a measure of underestimation of patient priority and assigning a subclass classification lower than required which can have catastrophic effects over time or inability to receive proper medical care.

To evaluate the compliance toward the policy a baseline audit was conducted in 2021 which recommended certain changes to be made in practice, which were implemented and then a reaudit was conducted in 2022 to re-evaluate if any improvements occurred in clinical practice. The audits have helped us reviewing and comparing results on appropriate triage categorization by nurses and compliance to recommended target times for attending the patients by physicians in primary health centers. The findings on prevailing practices have highlighted significant improvements in quality of care provided to the patients, which also leads to patients’ satisfaction. The findings of the audits are enumerated below.

## Methodology

### Data Collection

This was a multicenter interventional study conducted at 28 primary health care centers across Qatar. Electronic Medical records of patients presented at triage clinics were retrospectively reviewed from July 2021 to September 2021 for baseline examination. The data was stratified into 3 triage categories: Emergency, Priority, and Routine. Random sampling method was applied for each triage category. Medical record review and data collection were performed by clinical auditors by using an excel based tool developed based on audit criteria. Data was double checked and verified by the auditors before entering in the database.

A baseline audit was conducted by reviewing a representative sample of 554 triage health records from 28 heath centers in September 2021 against explicitly defined criteria on triage practices. The results showed practice gaps in appropriate triage categorization by nurses and physician seen time of patients within recommend target times. Resultantly, some key recommendations were made and implemented to improve compliance to triage walk-in policy.

In September 2022, a re-audit was conducted to reevaluate any changes in quality of triage practices post implementation of baseline audit recommendations by reviewing a representative sample of 470 medical records of patients seen at the triage room in 26 health centers from April 2022 to May 2022.

### Statistical Analysis

The relation between a nurse’s wrongful categorization and a delay in a physician’s seen time, and the relationship between a nurse’s right categorization and no delay in a physician’s seen time, were also studied by the auditors using correlation. In this investigation, a correlation coefficient of .94 was determined to be statistically significant.

Process mapping is a technique for outlining each stage in a process. It assists quality improvement teams in locating issues that might be resolved by simplifying a complicated procedure.^6^ A process map was used to illustrate the sequence of triage events, and the relationships between the activities which were examined to identify any potential delay in treatment.

**Figure 1.**
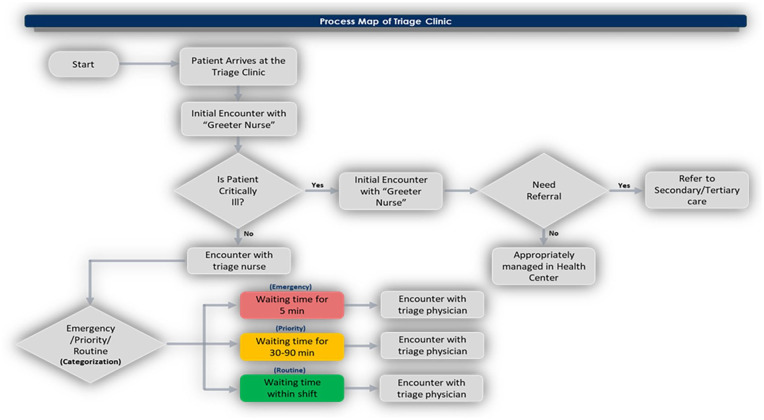
Triage clinic process map.

Figure 1 illustrates the triage clinic pathway and the relationship between each process. This helps identify possible causes for a problem.

### Intervention

According to the baseline audit findings, inappropriate triage categorization was one of the main reasons for the delay in the physician seen time in the triage clinic. Patients that required emergency attention had to wait for a lengthy period because of incorrect categorization. The audit team disseminated the audit results with the triage nurses to sensitize them on the gaps identified in their practice. Also, conducted CME (continuous medical educations), trainings/ lectures on the most common conditions/complaints in the triage and evaluation of competencies of nurses are expected to improve the triage categorization process.

During the evaluation of medical records, it was observed that the physicians did not provide for the overriding or modification of incorrect triage categorization. As a result of this the Physicians had missed an opportunity to reprimand and instruct the nurses on proper triage categorization. In coordination with the Clinical Information System (CIS) team, a directive/reminder was distributed to the physicians instructing them to utilize CIS features to change or override incorrect nursing categorization. Audit trails ensure monitoring of changes made in the CIS.

### Results

The reaudit findings depicted a rather mixed picture of improved compliance in some criteria and declined compliance in few other criteria compared to baseline. Evidence showed improvement in appropriate allocation of medical emergency triage category by nurses from 63% at baseline to 90% at the reaudit. In correlation with this change, the over triaging dropped from 37% in the baseline to 10% in the audit.

Compliance to the recommended time target of 5 min for physicians to attend medical emergency patients has shown marginal improvement from 48% in baseline to 55% in the re-audit.

There has been a drop in the proper patient allocation under the medical priority triage category from 91% at baseline to 74% at the reaudit, 26% of emergency cases were wrongly categorized as medical priorities.

Nonetheless, re-audit data showed that doctors had seen 100% of confirmed medical priority cases within the 90-min time target, which was also achieved 100% at baseline.

Compliance was slightly declined for appropriate triage allocation under medical routine from 100% at baseline to 95% in reaudit.

No evidence was found regarding changes/modification in nurses triage categories by the physicians, even though the CIS option to make such changes is available to them, denoting that they may be oblivious about it.

**Figure 2.**
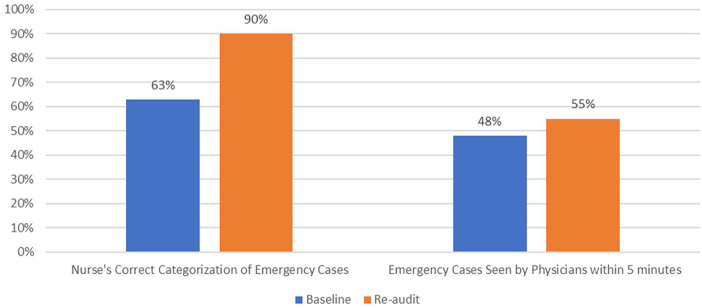
Appropriate categorization of medical emergency cases by nurses [N = 120 (baseline), N = 210 (re-audit)] and physician seen time [N = 75 (baseline), N = 190 (re-audit)].

Figure 2 shows that in reaudit substantial improvement occurred in triage-medical emergency categorization and marginal improvement was also seen in physician’s seen time compared to baseline.

**Figure 3.**
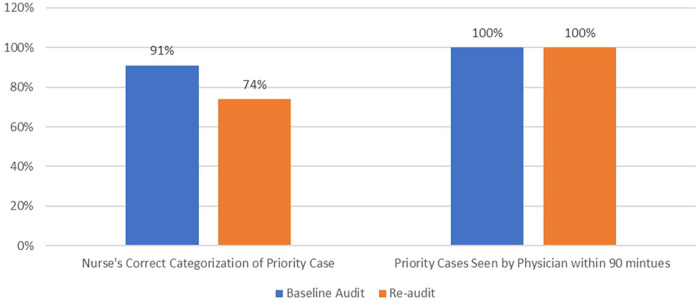
Appropriate categorization of medical priority cases by nurses [N = 222 (baseline), N = 130 (re-audit)] and physician seen time [N = 202 (baseline), N = 96 (re-audit)].

Figure 3 above shows appropriate categorization of medical priority cases, has slightly declined, however, physicians’ seen times remained unchanged compared to baseline.

**Figure 4.**
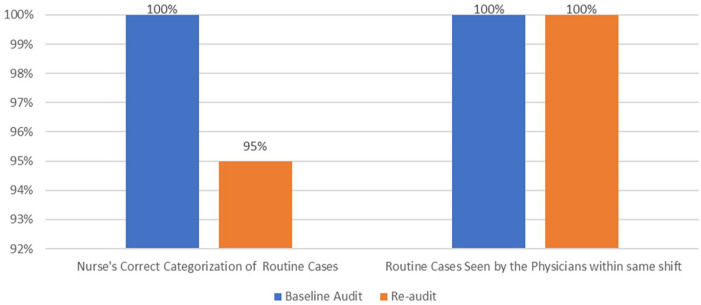
Appropriate categorization of medical routine cases by nurses [N = 212 (baseline), N = 130 (re-audit)] and physician seen time [N = 212 (baseline), N = 123 (re-audit)].

The above Figure 4 shows the categorization of medical routine cases declined slightly when compared to the baseline audit. However, physician-seen times has remained unchanged.

## Discussion

To identify patients who do not need earlier intervention and to make the best use of emergency medical resources for prompt treatment, accurate patient categorization is crucial.^7^ Our baseline audit had shown emergency case triage categorization had a suboptimal level of (63%) compliance, and it was observed that hardly in 48% of emergency situations the Physicians had attended the patients within the recommended 5 min. Studies showed that by correctly categorizing emergencies cases based on an accurate initial assessment of patients who attend the triage clinic, the length of time spent there can be decreased.^7^ Therefore, triage nurses in charge of initial assessment in the triage clinics should classify the severity of patients arriving quickly and accurately, which is deemed to be their primary role.^8^ Problem-based learning can improve the knowledge of triage nurses and boost the precision of triage categorization, which can subsequently improve the safety of the patient in the treatment room.^7^ Even though only 74% of patients were attended by the physicians within 90 min for priority cases, the categorization of triage priority cases showed 91% compliance. When routine cases were correctly categorized and all patients were physically seen during the same shift, it was determined that 100% compliance was the best level of compliance. Next step was to determine whether any relationship exist between nurses’ categorization and physicians’ seen time. A correlation analysis was performed to understand the relationship between nurse’s categorization and physician seen time

**Figure 5.**
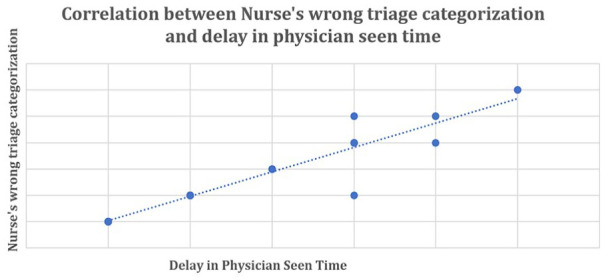
Relation between nurse’s incorrect categorization and delay in physician seen time for triage emergency and priority cases (N = 65).

Figure 5 elucidated there is a positive correlation between nurse’s wrong triage categorization and delay in physician see time (correlation co-efficient *r* = 0.94), which indicates that whenever the nurses are doing wrong categorization there has been a delay in the physician seen time.

**Figure 6.**
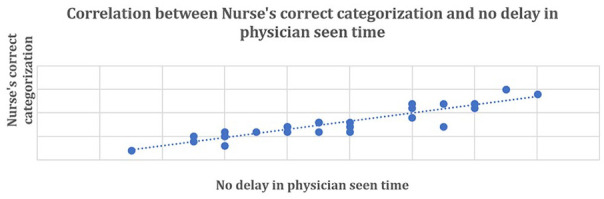
Relation between nurse’s correct categorization and no delay in physician seen time for triage emergency (75) and priority cases (202). N = 272.

Figure 6 shows there is a positive correlation between nurse’s correct triage categorization and no delay in physician seen time (correlation co-efficient *r* = 0.92), which indicates that whenever the nurses are performing triage categorization correctly there has been no delay in the physician seen time.

Correlation analysis showed existence of direct relation between proper/improper triage categorization and delay/no delay in physician seen time. Whenever wrong categorization occur physician seen time increase and vice versa. To understand the cause of wrong categorization and delay in physician seen time cause and effect analysis was carried out with help of fishbone diagram.

**Figure 7.**
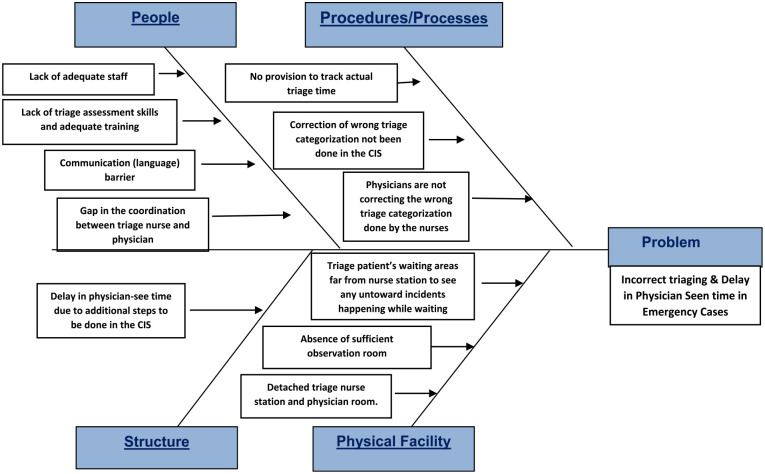
Fishbone diagram (cause and effect diagram).

Figure 7 shows a list of causes for incorrect triaging and delay in physician-observed time in emergency cases. This helps to select and fix the most feasible and important causes of a problem.

Ishikawa charts or fishbone charts are used to understand the multiple potential causes that contribute to quality-of-care problems and focus improvement efforts on those.^9^ We used a fishbone diagram with combination of 5 whys technique to reach a conclusion. Although, they were many possible causes identified, predominant causes noted was wrong triage categorization by nurses. Hence to decrease the amount of time it takes for a physician to see a patient, the triage classification process, carried out by nurses, must be primarily improved, as this is where first patient encounter happens. Although there is a comprehensive institutional triage policy which is made available that covers all areas of triage (such as emergencies, priorities, and routine cases), the classification of triage cases is inappropriate. Patient safety is related to the accurate categorization of high-urgency patients because incorrectly classifying high-urgency patients as low-urgency levels delays diagnosis and treatment, which may result in morbidity or mortality. Correctly classifying low-urgency patients improves Triage clinic workflow efficiency and decreases wait times for genuinely high-urgency Triage – Emergency visits.^10^ To mitigate deficiency of wrongful categorization, the audit team planned to train nurses on triage categorization. It has been demonstrated that triage refresher training improves categorization accuracy, lowering the likelihood of unfavorable patient outcomes.^11^ As part of training, continuous medical education (CME) was recommended, along with lectures on the most common conditions and complaints seen in the triage setting and to conduct competency tests to evaluate nurse skills To promote rectification of wrong triage categorization, audit had recommended to conduct awareness session for physicians on how to use CIS overriding option to modify triage categorization made by nurses if it deemed incorrect by the physicians. It was intended that such session might open an opportunity for discussion among physicians and nurses regarding incorrect triage categorization and to build consensus to prevent this type of error in the future. The audit report was also shared amongst all the staff, and this report was presented in dissemination meetings to create awareness.

After a period of 6 months, re-audit was conducted using the same criteria as of the baseline audit. The results of the re-audit showed an increase in the nurses’ categorization of emergency cases from 63% to 90%. This demonstrates how well the action plan was carried out. In a similar way, physician-seen time (within 5 min) of emergency cases has increased marginally from 48% to 55%. This illustrates the relativity between Physician seen time and case categorization as wrong categorization will lead to delay/increase waiting time for patient to be seen by the physician. Although the physician seen time has improved by just 7% since the baseline audit, this gain is still considerable when considering into account the number of health centers (28) included, the study population, and the threshold of 5 min for emergency cases. This improvement can be considered the hallmark of the action plan implemented. A similar study was conducted in Korea to confirm whether the triage categorization agreement among triage nurses could be improved through problem-based training and concluded that problem-based learning can improve the knowledge of triage nurses and improve the precision of triage classification, which subsequently improves the patient safety in the emergency room.^7^

The categorization of priority cases, however, fell from 91% to 74% in the re-audit. But the percentage of physicians seen in time was consistent at 100% with the baseline audit. Similarly, categorization of routine cases also fell from 100% to 95% in the re-audit; however, physician-observed time remained consistent at 100% with the baseline audit.

Although minor drop noticed in the categorization of priority and routine cases, physician seen time stayed constant. Significantly, 25% of incorrectly classified priority cases fell into the category of emergencies, while the remaining 1% were classified as routine cases. Emergency cases wrongly categorized as priority cases should be considered as under-triaging. Under-triaging will postpone the care of patients who require urgent care and lead to catastrophe. Making sure the right patient receives the appropriate care in the shortest period of time is essential in ensuring the patient’s safety; therefore, prioritization processes should be enhanced in order to accomplish this goal. The audit team suggested that the triage nurses continue their problem-based training until they reach the optimal level of compliance. It is also anticipated that with continued training, correct categorization of routine cases will be also improved.

## Limitation

We have discovered several causes during the cause-and-effect study for the delay in physician seen time. However, since further data revealed a significant correlation between triage categorization and physician-seen time, so our initial focus remained mainly concentrated on enhancing triage categorization. We will take into consideration the additional factors in the future re-audit as well.

## Future Directions

As quality-of-care improvement is a continuous process, some new recommendations have emerged from the reaudit which will be pursued onwards for implementation. Ongoing triage refresher training should be done on a regular basis, which has proven to improve overall triage practice.^12^ Enhancement of collaboration between triage physicians and nurses should be promoted for ongoing education and rectification. Use of technology like machine learning techniques will help in improving clinical prioritization to deliver better triaging in accordance with the Clinical Prioritization Criteria.^12^ Mandate all physicians to use the Clinical Information System option to make changes or modifications in the nurse’s triage categorization in case of the wrong categorization. Optimize documentation of patients under the emergency triage category at 3 points of contacts or encounters: with the greeter nurse, the triage nurse, and the physicians, by providing a practical solution to the triage team, for instance, a tablet with a bar code system for noting the real time of the patient journey during the triage process.

## Conclusion

In conclusion, this audit was primarily conducted to evaluate and comprehend the current state of the triaging practice in PHCC and to identify, any gaps to be focused for making improvements in quality of clinical practice as per the PHCC policy. The baseline audit allowed the audit team to intervene and enhance the practice by highlighting its shortcomings as well as its strengths. One of the main reasons for the triage clinic’s physician seen time delays was improper triage categorization. The results of the reaudit were encouraging and confirmed that the baseline recommendations proved to be useful to make some improvements in the quality of the triaging process in PHCC; however, there is room for further improvement. As for patients to receive emergency care at the most suitable time, a precise triage decision is necessary. To acquire this triage decision, the reaudit recommends that triage nurses’ skills should be further boosted and continually improved. The audit team proposed problem-based training to be arranged for nurses. Re-audit showed significant improvement in the correct triage categorization and correspondingly physician-seen time of emergency cases. The audit team has good grounds to think that this improvement is connected to ongoing triage training.

Physician leads who are well-versed in the triage system should plan and provide the triage process related instructions to their teams at each health center. A collaboration between triage physicians and nurses is inevitable for ongoing corrective and preventive action. To determine need for triage training as soon as feasible, a monthly surveillance is advised, and appropriate triage accuracy should be developed. Early detection of inaccurate triage would enable team to take an early intervention to reduce the frequency of unfavorable events in the triage clinics. As a result, overall performance of adhering to PHCC policy for triage process may be improved.

Since there aren’t many studies on triage training and its relation to physician seen time, more studies are advised to be conducted to discover and assess how well triage training can increase triage accuracy and reduce physician see time.
